# *In vivo* mutation rates and the landscape of fitness costs of HIV-1

**DOI:** 10.1093/ve/vex003

**Published:** 2017-03-02

**Authors:** Fabio Zanini, Vadim Puller, Johanna Brodin, Jan Albert, Richard A. Neher

**Affiliations:** 1 Max Planck Institute for Developmental Biology, Tübingen 72076, Germany; 2 Department of Bioengineering, Stanford University, Stanford, CA 94305, USA; 3 Department of Microbiology, Tumor and Cell Biology, Karolinska Institute, SE-171 76 Stockholm, Sweden; 4 Department of Clinical Microbiology, Karolinska Institute, SE-171 76, Stockholm, Sweden

**Keywords:** Evolution, HIV-1, mutation rate, fitness landscape.

## Abstract

Mutation rates and fitness costs of deleterious mutations are difficult to measure *in vivo* but essential for a quantitative understanding of evolution. Using whole genome deep sequencing data from longitudinal samples during untreated HIV-1 infection, we estimated mutation rates and fitness costs in HIV-1 from the dynamics of genetic variation. At approximately neutral sites, mutations accumulate with a rate of 1.2 × 10^−5^ per site per day, in agreement with the rate measured in cell cultures. We estimated the rate from G to A to be the largest, followed by the other transitions C to T, T to C, and A to G, while transversions are less frequent. At other sites, mutations tend to reduce virus replication. We estimated the fitness cost of mutations at every site in the HIV-1 genome using a model of mutation selection balance. About half of all non-synonymous mutations have large fitness costs (>10 percent), while most synonymous mutations have costs <1 percent. The cost of synonymous mutations is especially low in most of *pol* where we could not detect measurable costs for the majority of synonymous mutations. In contrast, we find high costs for synonymous mutations in important RNA structures and regulatory regions. The intra-patient fitness cost estimates are consistent across multiple patients, indicating that the deleterious part of the fitness landscape is universal and explains a large fraction of global HIV-1 group M diversity.

## 1. Introduction

HIV-1 evolves rapidly within individual hosts and accumulates mutations that allow the virus population to evade immune recognition. Mutations arise during reverse transcription, during forward transcription by the human RNA polymerase II, or through mutagenesis by host factors (Mansky and Temin 1995; O’Neil et al. 2002; Malim 2009; Abram et al. 2010; Smyth et al. 2012; Cuevas et al. 2015; ). Characterization of the mutation rate and the genome wide landscape of fitness effects is a prerequisite for a quantitative understanding of the evolutionary dynamics of HIV and for rational design of both vaccines and resistance-proof antiretroviral drugs.

The frequencies of *de novo* mutations during HIV-1 replication have been studied *in vitro* using cell culture systems (Mansky and Temin 1995; O’Neil et al. 2002; Abram et al. 2010); a total rate of about 2 × 10^−5^ mutations per site per replication cycle is reported. Recently, Cuevas et al. (2015) reported a much higher mutation rate *in vivo*, but that study focused on integrated provirus and might not reflect the mutational frequency in the circulating HIV-1 virions. To bridge these conflicting perspectives, we estimated the frequency of *de novo* mutations in circulating HIV-1 RNA within untreated patients.

Of all mutations that are generated daily within an HIV-1 positive individual, the majority decrease virus replication, some mutations are neutral and have little or no effect, and a minority of mutations are beneficial to virus replication. While beneficial mutations rapidly spread through the virus population within a patient, deleterious mutations stay at low frequency in a balance between mutation and selection. Beneficial mutations are often patient-specific, for example because they mediate escape from cytotoxic T-lymphocytes (CTL) and neutralizing antibodies (Goonetilleke et al. 2009; Bar et al. 2012; Walker and McMichael 2012). Most mutation, including immune escape mutations, lower intrinsic viral fitness: host-specific adaptation is a trade-off between immune evasion and the intrinsic fitness costs of escape mutations.

The cost of individual mutations can be quantified by competing mutant and wild-type viruses in cell culture (Parera et al. 2007; Martinez-Picado and Martinez 2008). Similar measurements of replication capacity are done routinely for drug resistance testing (Petropoulos et al. 2000) and have been used to infer the fitness landscape of the HIV-1 protease and reverse transcriptase (Hinkley et al. 2011). Recently, high-throughput methods have been developed to systematically identify the amino acid preferences or fitness costs at every position in a protein (Acevedo et al. 2014; Thyagarajan and Bloom 2014; Rihn et al. 2015; Haddox et al. 2016). Fitness landscapes have also been estimated indirectly from large global collections of sequences (Dahirel et al. 2011; Ferguson et al. 2013). These methods assume that high fitness variants are at high frequency in the global HIV-1 population. Either approach has limitations: Cell culture experiments are not sensitive to small costs since a large number of passages are necessary to observe small fitness costs. Models based on cross-sectional data are confounded by immune escape because they cannot differentiate between diversifying selection by the immune system and the absence of functional constraints.

In contrast to immune escape mutations, the landscape of intrinsic fitness costs is expected to be similar across different HIV-1 isolates. However, the effect of a particular mutation can depend on other sites in the genome—a phenomenon known as epistasis—which can result in different fitness costs on different genetic backgrounds (de Visser and Krug 2014). Such interactions between mutations have been observed as compensatory evolution after CTL escape (Schneidewind et al. 2009) or as covariation of amino acids (Carlson et al. 2008; Dahirel et al. 2011). Since sequences of the same HIV-1 subtype differ at only about 10 percent of amino acids (Li et al. 2015), the majority of residues with which a given amino acid interacts will be conserved and the fitness effects of mutations are expected to be similar across HIV strains. Similarly, Doud et al. (2015) have shown that the majority of mutation effects tend to be conserved in mildly diverged influenza virus proteins.

Here, we estimate the rates and spectrum of mutations and the landscape of fitness costs of HIV-1 using whole genome deep-sequencing data from longitudinal samples (Zanini et al. 2015). We first use the accumulation of natural divergence at a subset of approximately neutral sites to estimate the *in vivo* mutation rates between all pairs of nucleotides. We then determine fitness costs of mutations away from the HIV-1 group M consensus sequence from the *in vivo* intra-patient balance of mutation and selection against deleterious variants. Our cost estimates are most sensitive for small and moderate costs (between 0.1 and 10 percent), not affected by patterns of immune escape, and not restricted to one single protein: we estimated fitness costs at almost every position of the HIV-1 genome. We then investigate signatures of RNA structure elements or biophysical properties of HIV-1 proteins in the genome wide landscape of fitness costs and study fitness costs at sites associated with CTL selection or drug resistance.

## 2. Materials and methods

### 2.1 Study patients and data sources

We analyzed longitudinal whole genome deep sequencing data from nine HIV-1 patients described in Zanini et al. (2015) and an additional patient p7 that was described by Brodin et al. (2016). A summary of patient characteristics is given in Supplementary Table S1. The first sequenced sample was within 7 months of infection for all patients other than p7. Genetic diversity within this first sample suggested that the virus population in all patients other than p3 and p10 are dominated by a single founding genotype (Zanini et al. 2015) which we approximate by the consensus sequence of the first sample.

The HIV genome was amplified in six overlapping fragments of ∼2 kb. Each of these amplicons was sequenced to high coverage on a MiSeq instruments with 2 × 250 bp reads. The median number of reads per amplicon was 80,000 (quartiles 20,000–220,000, max 2 millions). For a detailed summary of the sequencing statistics, see Zanini et al. (2015). For each patient, coverage, divergence from the founder virus strain, and diversity are reported in the original publication as well as online at the web page http://hiv.tuebingen.mpg.de. The sequencing reads are available in the European Nucleotide Archive under project accession number PRJEB9618.

The nucleotide and amino acid cross-sectional alignments of HIV-1 group M were downloaded from the Los Alamos National Laboratory HIV database and filtered for short or otherwise problematic sequences and are available as Supplementary Material.

Disorder and solvent accessibility scores amino acids for different HIV proteins were provided by the authors of Li et al. (2015) (available at www.virusface.com). These scores were mapped to homologous positions in the virus populations via alignments to the reference sequence NL4-3. Positions without scores were discarded.

### 2.2 Theoretical background

The basic quantity that we track in this article is the frequency of single nucleotide variants (SNVs), which we also call ‘alleles’. Given a certain allele is generated by mutation at a rate *μ* and bears a logarithmic fitness cost *s*, its frequency in the viral population *x* is described by (Haldane 1937; Haigh 1978):

(1) $$\frac{d}{dt}x(t)=\mu -sx(t)+\xi (x,t).$$

The noise term ξ(x,t) models stochastic evolutionary processes, including genetic drift and hitchhiking. If recombination is rapid and selected SNVs are rare, hitchhiking is negligible and simple genetic drift is the dominant contribution to *ξ*. In this case, the equilibrium distribution of *x* can be calculated via Kimura’s diffusion theory (Kimura 1964). At lower recombination rates and high density of selected SNVs, as is the case in intra-patient HIV-1 evolution (Neher and Leitner 2010; Zanini et al. 2015), the stochastic dynamics of *x* is much more complicated. However, in this article, we are only concerned with the *mean* allele frequency *x*. In contrast to higher moments of *x*, the *mean* does not depend on properties of *ξ* since Eq. 1 is linear. An intuitive explanation for this behavior is that a positively selected allele can be linked to any one of the four nucleotides at the hitchhiker’s position, without preference.

The average frequency of SNVs with fitness cost *s* is given by:

(2) $$\langle x\rangle =\frac{\mu }{s}(1-e^{-st})\;\;\;\;\text{for}\;\;\;\;s>0,$$

(3) 〈x〉=μt     for s→0.

Approximately neutral alleles with s≈0 accumulate linearly and we will use this behavior to estimate the mutation rates of HIV-1. The frequency of alleles under purifying selection with *s* > 0 saturates at $\bar{x}=\mu /s$ after a time of order $s^{-1}$ (Haldane 1937). The fitness cost *s* can be estimated both from the approach to saturation and the level of saturation μ/s. This approach has been generalized to complex fitness landscapes (Seifert et al. 2015).

Note, however, that Eq. 2 only holds on average and suitable ensembles, that is, sets of sites with similar properties, need to be defined and averaged to leverage (Eq. 2). We define and use two such ensembles (‘Sat’ and ‘Pooled’) below.

### 2.3 Data processing

The sequencing reads from the longitudinal samples were analyzed using the library hivevo_access, available at https://github.com/neherlab/HIVEVO_access. The analysis scripts used for this paper, as well as the resulting data for the mutation rate and fitness cost estimates, are available online at: https://github.com/iosonofabio/HIV_fitness_landscape.

The counts of each of the four nucleotide at each genomic position and for each sample were normalized to obtain frequencies and corrected to eliminate spurious diversity caused by RT-PCR and sequencing errors. In our test experiments with homogeneous HIV-1 samples (Zanini et al. 2015), we observed that almost none out of several thousand genomic positions had an error rate above 0.2 percent and we defined this as a conservative threshold for background noise. For every plasma sample, all frequencies below this threshold were set to 0.

### 2.4 Mutation rate estimation

We estimate mutation rates from the linearly increasing divergence at approximately neutral sites in patients where the initial sample was almost homogeneous with no evidence of infection by multiple virions. For each patient, we selected a set of approximately neutral positions in the HIV-1 genome at which (i) the entropy in a group *M* alignment is higher than 0.3 bits and (ii) the consensus nucleotide of the earliest sample is equal to the HIV-1 group *M* consensus at this position. Derived variants at those sites are considered if (i) they are translated in a single reading frame, (ii) they are synonymous changes, (iii) they are outside of known RNA structures or overlapping reading frames. The protein *gp120* has been shown to be sensitive to synonymous mutations and recoding (Zanini and Neher 2013; Vabret et al. 2014), but inclusion or exclusion of *gp120* did not make a difference.

**Figure 1.**
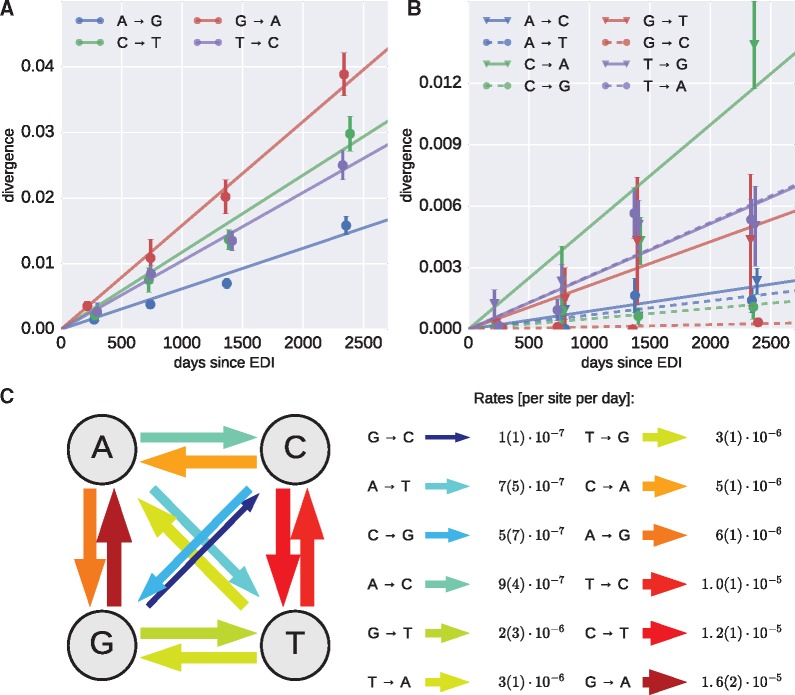
Mutation rate estimates. (A, B) Accumulation of divergence at approximately neutral sites for transitions and transversions, respectively (EDI: estimated date of infection). The slope of the individual regression lines in panels A and B provide estimates of the *in vivo* mutation rates. (C) Schematic representation and quantification of the mutation rates. Error bars for the estimates, indicated in parenthesis as uncertainties over the last significant digit, are standard deviations over 100 patient bootstraps.

The frequencies of these synonymous changes are grouped by mutation (e.g., A→G) and averaged across the genome. We further bin samples by their time since the Estimated Date of Infection (EDI) in the bins of [0, 500, 1000, 1750, 3000] days. The time-binned average frequencies are modeled by a linear fit with zero intercept, so the inferred rate $\hat{\mu }$ is:

(4) $$\hat{\mu }=\frac{\sum_{i}t_{i}\cdot x_{i}}{\sum_{i}t_{i}^{2}},$$

where (*t_i_*, *x_i_*) are the center and average divergence of bin *i* (see Fig. 1A and B). The rates of mutations between all pairs of nucleotides are estimated independently to obtain the complete matrix of mutation rates. The whole procedure is repeated for 100 bootstraps over patients to estimate the uncertainty of the rates, shown as errors in Fig. 1C. Variations of the inclusion criteria have been tested and yielded similar results, see Supplementary Fig. S2.

### 2.5 Estimation of selection coefficients

We used Eq. 2 in two complementary ways to estimate fitness costs. The ‘Sat’ method groups sites in the genome by cross-sectional conservation and estimates the harmonic average of *s* at those sites from the accumulation of intra-patient diversity. The ‘Pooled’ method estimates fitness costs of non-consensus alleles at individuals positions in the HIV genome by combining all measurements from all samples into a single estimate.

### 2.6 Average fitness costs from divergence saturation

For seven out of the ten patients, early samples are available and there is no evidence of multiple founding viruses. In these patients, we expect divergence to increase on average according to Eq. 2.

Since individual frequency trajectories *x*(*t*) are noisy, we need to average $x_{i}(t)$ over many sites *i* with similar *s* before comparing Eq. 2 to data. To identify a set of sites with similar *s*, we used conservation as a proxy for fitness cost and grouped all positions in the genome by their conservation in a representative group *M* alignment and fit (Eq. 2). Furthermore, we considered only sites at which (i) the majority nucleotide at the earliest time point equals the global HIV-1 group *M* consensus and (ii) the majority amino acid does not change during the infection. The latter criterion is necessary to exclude sites under positive selection, for example, because they mediate immune escape of revert previous immune escape mutations. Instead of modeling the mutations and fitness costs of all four nucleotides, we used a simplified 2-state model: the group *M* consensus state and the sum of the derived mutations.

To fit Eq. 2 to the data, we bin all samples by EDI and minimize the squared deviation between Eq. 2 and the average divergence in these bins with respect to *s* while keeping $\mu =1.2\times 10^{-5}$ /day constant. By fitting Eq. 2 to average diversity, one estimates harmonic averages of fitness costs of non-consensus nucleotides in the different entropy categories.

In the Supplementary material, we present another method to estimate fitness costs from average SNV frequency data that exploits correlations between SNV frequencies at successive times points. We found the results to be comparable to the simpler method discussed above.

### 2.7 Site-specific fitness cost estimates

For $t\gg s^{-1}$, Eq. 2 tends to $\bar{x}=\mu /s$. After 2 years, frequencies at sites with fitness costs as low as $s_{i}=0.002$ are expected to be close to equilibration and the frequencies of these mutations fluctuate around ${\bar{x}}_{i}$. If the saturation frequency ${\bar{x}}_{i}$ at position *i* can be accurately measured, site-specific fitness costs can be estimated via $s_{i}=\mu /{\bar{x}}_{i}$. To obtain accurate estimates of ${\bar{x}}_{i}$, we averaged SNV frequencies at individual sites over all plasma samples that were taken more than 2 years after infection from all patients.

As before, we exclude sites at which the initial consensus does not agree with the global HIV-1 consensus and sites that sweep (i.e., where the majority state changes during infection). These exclusions are particularly important, since sites from different patients are combined and minor frequencies are only meaningful when measured relative to the same reference nucleotide or amino acid.

In each sample, the accuracy at which we can measure *x_i_* is limited by sequencing errors, and more importantly by the often small number of HIV-1 RNA molecules that contribute to each sample (Zanini et al. 2015). Hence, a rare SNV will only be observed in a fraction of samples. However, the average SNV frequency across samples reflects the true frequency and by combining many samples the accuracy of our estimate of ${\bar{x}}_{i}$ can be pushed below the error threshold in a single sample. If, for example, an SNV is observed in 10 percent of samples at frequency 0.5 percent (possibly a single template) and not observed in 90 percent of samples, the average frequency of this mutation would be estimated to be ∼0.05 percent. Because more template molecules were captured in some plasma samples than in others—we estimated the number of templates by limiting dilution, see Zanini et al. (2015)—we perform a weighted average: for each patient, the average frequency of nucleotide or amino acid *α* at position *i* is then given by

(5) $${\hat{x}}_{i,\alpha }=\frac{1}{\sum_{k}w_{k}}\sum_{k=1}^{n}w_{k}x_{k,i,\alpha },$$

where $x_{k,i,\alpha }$ is the frequency in sample *k* and the sum runs over all samples k=1,…,n that are at least 2 years after infection. The weight *w_k_* is calculated from the estimated number of template molecules *T_k_* as $w_{k}={\left(0.002+1/T_{k}\right)}^{-1}$, where 0.002 is the combined error rate of RT-PCR and sequencing (see above). After this weighing, samples contribute proportionally to the number of RNA templates when *T_k_* is small, while for large *T_k_* the sequencing error rate is limiting and the per sample contribution is capped at 500. After averaging samples within patients, we average ${\hat{x}}_{i,\alpha }$ over patients and sum all non-consensus nucleotides or amino acids to obtain the average non-consensus frequency ${\hat{x}}_{i}$ for each position *i* in the HIV-1 genome. The fitness cost at position *i* is then estimated by $\mu_{i}/{\hat{x}}_{i}$ where *µ_i_* is the mutation rate away from the consensus nucleotide at position *i*. To determine the uncertainty of fitness cost estimates, we picked sites within small slices of the distribution of selection coefficients and constructed distributions of fitness cost estimates at these sites through bootstrapping over patients.

Estimates of fitness costs for nucleotide and amino acid mutations were done in analogous ways but amino acid mutation rates are calculated specifically for each patient on the bases of the triplet encoding for the amino acid in the founder sequence of that patient (amino acid changes requiring two nucleotide changes were ignored).

## 3. Results

### 3.1 The rate and spectrum of mutations in HIV

To estimate mutation rates from longitudinal and deep whole genome sequencing data, we identified a set of positions at which mutations are approximately neutral and exploited the fact that the rate of divergence at neutral sites is precisely the *in vivo* mutation rate (see Methods and Kimura 1968). Figure 1A and B shows the average divergence from the approximate virus founder sequence in this approximately neutral set, for all twelve nucleotide substitutions. We pooled data from patients p1, p2, p5, p6, p8, p9, p11 (those with early samples and without suspected dual infection); the error bars indicate standard deviations over patient bootstraps. The data confirm that divergence increased linearly, suggesting that positions under weak purifying selection did not dominate the set of sites selected by the above criteria. We estimated the mutation rates between each pair of nucleotides by linear regression—indicated by straight lines. Transition rates are 5-fold higher than transversions, while the total mutation rate per site is about 1.2 × 10^−5^ per site and day. The highest rate is G→A, while the lowest rates are transversions between Watson–Crick binding partners. The smallest rates cannot be measured accurately because the corresponding mutations are hardly observed. If the approximately neutral set contained a fraction of constrained sites, our method would slightly underestimate the rates without affecting our general conclusions. Positive selection at synonymous sites is unlikely to be common and a small number of such sites would not change our estimates substantially.

### 3.2 Landscape of fitness costs in the HIV-1 genome

While divergence at neutral sites increases linearly with time, purifying selection results in slower divergence and saturation of minor SNV frequencies (Eq. 2). We exploit this saturation of divergence to estimate fitness costs.

### 3.3 Relationship of global sequence conservation and fitness costs

**Figure 2.**
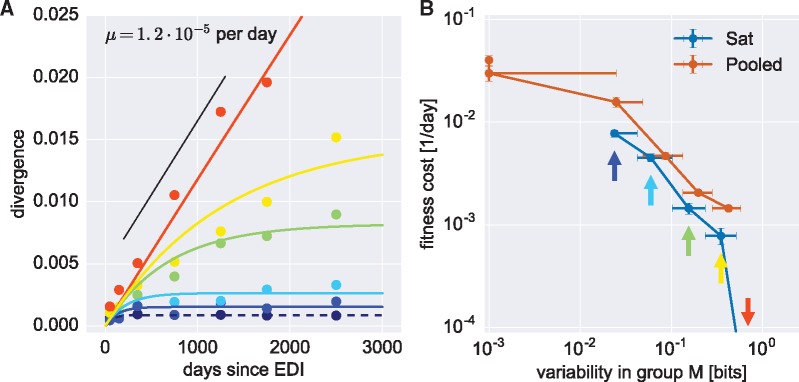
Average intra-patient fitness cost across quantiles of global HIV-1 group M diversity. (A) Divergence (measured as 1 − frequency of the ancestral state) saturates fast in the conserved parts of the genome (dark blue to cyan), more slowly in regions of intermediate conservation (green and yellow) and keeps increasing at the least conserved sites (red dots). The solid lines show fits of Eq. (2) to the binned data with fitness cost *s* as free parameter while the mutation rate is fixed at 1.2 × 10^−5^ per site per day (black line). (B) The “Sat” line shows fitness cost estimated for the blue, cyan, green, yellow, and red curves of panel A (indicated by arrows of the same colors). The most conserved quantile (dashed dark blue line in panel A) is not shown because saturation happens too rapidly to obtain an accurate fit. The “Pooled” line refers to harmonic averages of site-specific cost estimates. The ranges of entropy values contributing to each data point are indicated by horizontal lines, while the vertical error bars refer to the standard deviation of 100 bootstraps over patients: note that while error bars are small, there is substantial variation of fitness costs across sites *within* each diversity group.

To a first approximation, conservation of a site across many HIV-1 isolates is expected to be a proxy for high fitness cost of mutations, while mutations at a site that is observed in many different states probably have little or no fitness cost. To quantify the relationship between conservation and fitness cost *s*, we sorted sites in the HIV-1 genome into six groups of equal size and increasing global diversity (measured by Shannon entropy of columns in an alignment of group M sequences, see “Materials and methods”). Instead of estimating fitness costs for all three possible mutations at a given site, we estimated one fitness cost parameter for each site as the cost of the typical mutation away from the global consensus sequence (a more elaborate model that includes the twelve different mutation rates is described in Supplementary Fig. S3). For each conservation group, we average the frequencies of non-consensus nucleotides over all sites and patient samples in seven time bins. These average divergences are indicated by dots in Fig. 2A along with a nonlinear least square fit of Eq. (2) to the data of each group (each color indicates a conservation group, blue to red by increasing diversity). We set μ = 1.2 × 10^−5^ per site per day according to our estimate of the neutral mutation rate and fit a single parameter, the fitness cost *s*, for each group. The least conserved group accumulates divergence linearly at a rate that is consistent with the mutation rate estimate, while divergence saturates more rapidly and at lower levels with increasing conservation.

The estimated average costs and their error bars from 100 bootstraps over patients are shown in Fig. 2B as a blue line (“Sat”). The fitness cost of mutations in the least conserved 1/6 of the genome is undetectably small, consistent with neutrality. More conserved sites have higher costs, up to ∼1 percent for sites where the group M alignment entropy is ∼0.03 bits. For even more conserved sites (dashed line in Fig. 2A), saturation is very fast and we estimated the fitness cost using a different averaging procedure (see below).

Notice that for Eq. (2) to hold, it is essential that the infection is dominated by a single founder sequence. For this reason, patients p3 and p10 were excluded from this part of the analysis since our data indicate that they were infected by more than one viral variant. Furthermore, it is important to exclude sites subject to immune selection and sites where the initial nucleotide differs from the global consensus. Otherwise, rapid rise of beneficial mutations driven by CTL escape or reversion increase divergence and result in underestimation of the fitness costs.

### 3.4 Site-specific fitness costs in the HIV-1 genome

In addition to averaging mutation trajectories across multiple sites, we also estimated site-specific fitness costs by averaging data from multiple plasma samples during late infection. Average frequencies at sites where mutations carry large costs saturate rapidly after a time 1/*s*. Frequencies of minor variants in different samples are therefore uncorrelated and can be averaged to increase the accuracy of frequency estimates which then allows direct estimation of site specific costs *s_i_* from the relation ${\bar{x}}_{i}=\mu /s_{i}$, see “Materials and methods”.

**Figure 3.**
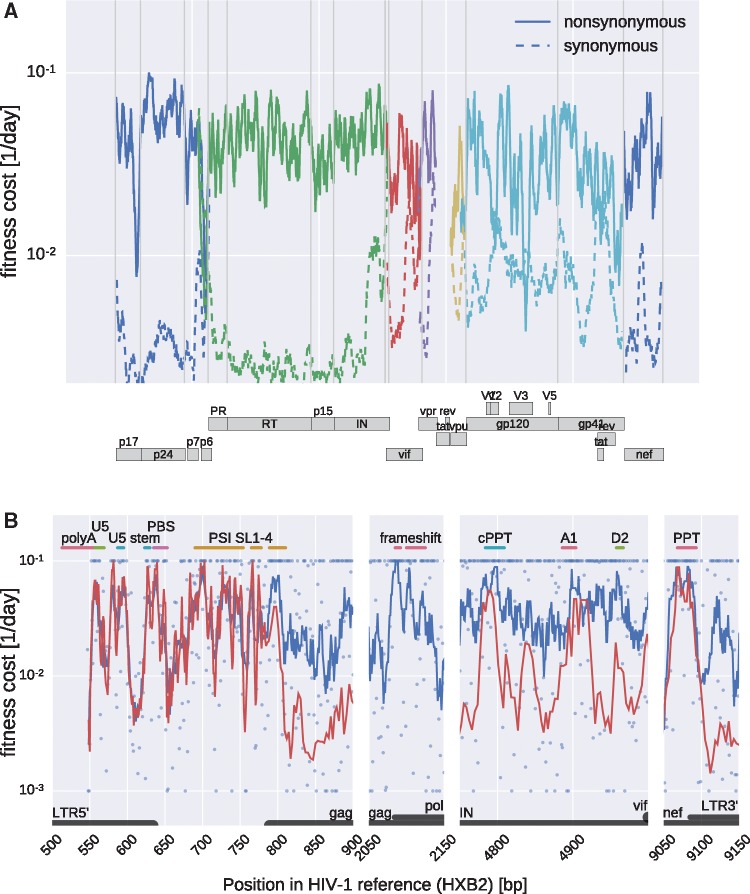
Fitness costs along the HIV-1 genome. (A) Fitness costs of synonymous and non-synonymous mutations in *gag*, *pol*, *vif*, *vpu*, *env*, and *nef* as a geometric sliding average with a window size of 30 bases. Estimates in *gp120* are expected to be less accurate due to consistent difficulties amplifying this part of the genome. (B) Fitness costs in selected regions of the genome that contain important regulatory elements. Blue dots show estimates for individual bases, blue lines indicate running averages with a window size of eight bases and red lines are running averages excluding bases where mutations cause amino acid changes. PBS: tRNA primer binding site. U5: unique 5′ region. SL 1–4 PSI: stem loops of the PSI packaging signal. (c) PPT: (central) poly purine tract. A1, D2: splice sites.

Figure 3A shows fitness costs of mutations at most positions along the HIV-1 genome (including *env*) separately for synonymous and non-synonymous mutations: the numerical estimates for all sites are available in the Supplementary Materials. The costs of synonymous and non-synonymous mutations are clearly different. Before analyzing these patterns in details (see below), as a consistency check we compared in Fig. 2B the average estimates (“Pooled” line) to our previous estimates “Sat”, which take into account the explicit time information of the samples. We found good agreement between the two approaches. We determined the uncertainty of fitness cost estimate by bootstrapping over patients in all major genes of HIV-1, see Supplementary Fig. S5. The variation is approximately twofold in each direction, so fitness costs above 5 percent are clearly separated from costs of 1 percent or less.

Fitness costs at single sites estimated from within patient diversity data anti-correlate strongly with global HIV-1 group *M* diversity (rank correlation ρ≈−0.7 for per site diversity measured by entropy, see Supplementary Fig. S4). Importantly, a particular site contributes to the estimate only if the founder and majority nucleotide in that sample equals the consensus variant. This condition removes any direct signal of cross-sectional diversity. The correlation increases as intra-patient variation is estimated using more patients (see Supplementary Fig. S4), suggesting that fitness costs at individual sites is largely conserved between patients. Supplementary Fig. S4 also shows scatter plots of global diversity vs fitness costs.

### 3.5 Distributions of fitness costs

**Figure 4.**
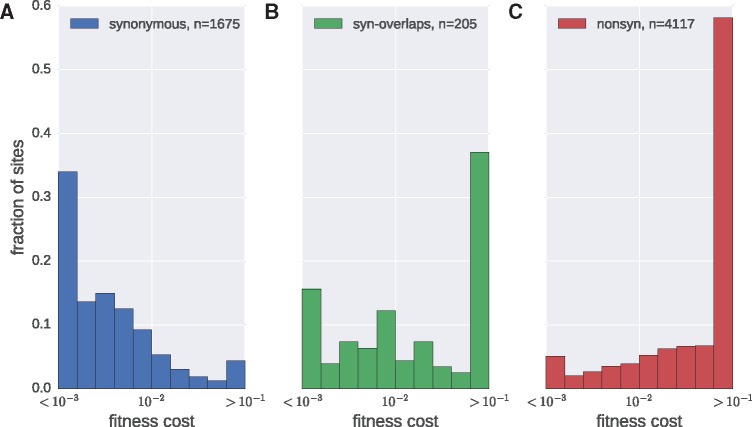
Distributions of fitness costs within coding regions. (A) Synonymous mutations, (B) mutations that are synonymous in one gene but affect another protein in a different reading frame, and (C) non-synonymous mutations (includes codons in *gag*, *pol*, *vif*, *vpu*, *vpr*). Half of non-synonymous mutations are very costly (>10 percent), while most synonymous mutations have a relatively small cost (<1 percent). The extremal bins include all points beyond the axis boundary. Fitness costs are measured in 1/day.

We observe marked differences between the distributions of fitness costs of synonymous and non-synonymous mutations (see Fig. 4): about half of all non-synonymous mutations have estimated fitness costs in excess of 10 percent, while the majority of synonymous mutations have fitness costs below 1 percent. The distribution of fitness costs of mutations that are synonymous in one gene but that affect another gene in a different reading frame resembles that of non-synonymous mutations (see Fig. 4B). We estimate ∼10 percent of synonymous mutations outside *env* to be highly deleterious; we discuss the specific costs of synonymous mutations in more detail below.

Supplementary Fig. S6 shows the distribution of fitness costs for different genes. In *gag* and *pol*, the contrast between synonymous and non-synonymous mutations is greatest. Synonymous mutations are costly in several isolated regions discussed below but have low fitness effects in much of *pol* and *gag*.

### 3.6 Fitness costs at functional RNA elements

The HIV-1 genome contains a number of well-characterized RNA elements that regulate different stages of the replication cycle. Many of these elements are embedded in protein-coding sequence and have been shown to reduce synonymous diversity (Ngandu et al. 2008; Mayrose et al. 2013). Indeed, in Fig. 3B important regulatory elements are clearly visible as well-defined peaks in the running averages of fitness costs along the genome.

In the 5′ LTR the largest fitness costs overlap with the hairpin containing the poly-A signal, the U5 sequence (Lu et al. 2011), the base of the following hairpin, the primer binding site (PBS) and stems 1–4 of the PSI element (LANL HIV sequence data base 2016). The frameshift region (slippery sequences plus hairpin), the splice acceptor site A1, and the polypurine tracts (PPT) in integrase and at the 3′ LTR show similarly high fitness costs (the TAR element is only partially covered by the sequencing data set and hence not shown here).

Mutations within the fourth stem loop of PSI at the beginning of *gag* are almost never observed, while synonymous sites are almost free to vary beyond the end of the stem. Synonymous mutations in the RRE are costly, but not as deleterious as those in PPT, the splice acceptor site A1, or the PSI element, indicating a higher evolutionary plasticity. Beyond these known elements, the correlation of fitness costs at synonymous mutations with cross-sectional diversity (Supplementary Fig. S9) suggests that there are a number of additional regions that might have important function on the nucleotide level, for example a few narrow peaks in *pol*. While well-characterized RNA elements correspond to clear patterns in the estimated fitness costs, RNA secondary structure predictions correlate poorly with fitness costs (see Supplementary Fig. S9 and discussion below).

### 3.7 Fitness costs and immune selection

Among sites that are globally variable (Shannon entropy above 0.1 bits), non-synonymous mutations are much more likely to have a high fitness cost (> 0.03 per day, odds ratio 15). This enrichment is most pronounced in *pol*, *gag*, and *nef* with little enrichment in *env*. This observation is consistent with host-specific selection pressures (CTL selection) at sites with large fitness costs. The resulting adaptations contribute to global diversity but revert quickly when transmitted to a new host (Friedrich et al. 2004; Leslie et al. 2004; Li et al. 2007; Zanini et al. 2015).

**Figure 5.**
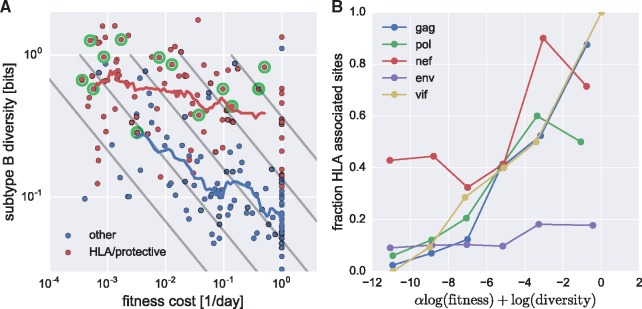
CTL selection blurs the relationship between fitness costs and diversity. (A) Each dot represents a site in *nef*: red (blue) dots are associated (not associated) with HLA types (Carlson et al. 2012). Dots surrounded by a green circle are associated with low viral load (Bartha et al. 2013). Intrapatient fitness costs are anticorrelated with subtype diversity (Spearman ρ=−0.59). The majority of sites in *nef* with high diversity despite high fitness costs—top right corner—are associated with either HLA types or with low viral load, while few sites in the lower left corner are associated with HLA variation. Panel B quantifies this trend by plotting the fraction of HLA associated sites in bins of increasing diversity and fitness costs (bin boundaries are denoted by straight grey lines in panel A, *α*  = 2). This figure uses data from subtype B patients only.

Such patient-specific selection has the potential to blur the relationship between fitness cost and diversity, as shown in Fig. 5A for *nef* (see Supplementary Fig. S4 for other genes). The majority of sites with high fitness costs and high cross-sectional diversity (upper right corner of Fig. 5A) have been reported to be associated with host HLA type (Carlson et al. 2012, shown in red) or with low viral load (Bartha et al. 2013, annotated dots). HLA-associated sites that fall into the top right corner of Fig. 5A are of particular interest since they are expected to result in virus control if targeted by strong CTL responses (Pereyra et al. 2014).

To quantify the overrepresentation of HLA-associated sites among diverse positions where mutations incur large fitness costs, we plotted the fraction of HLA-associated sites in bins indicated by diagonal straight lines in Fig. 5A for the genes *gag*, *pol*, *vif*, *env*, and *nef*. Bin boundaries are defined by α log ⁡(fitness)+ log ⁡(diversity)=const. with *α*  = 2. For all genes other than *env*, the fraction of HLA-associated sites increases strongly in bins corresponding to high diversity and fitness cost indicating that CTL selection pressure is responsible for global diversity that is deleterious to virus replication.

Notice that HLA-associations can only be detected for sites with some global variation. Hence, there is a strong ascertainment bias and almost all HLA-associated sites are found in the top half of Fig. 5A. Without independent characterization of this bias, a statistical assessment of the relation between CTL selection pressure, fitness cost, and global diversity remains challenging.

### 3.8 Fitness costs are weakly correlated with protein disorder and solvent accessibility

Perturbations to protein structure are expected to reduce virus fitness. Hence, mutations that decrease protein stability, occur in tightly packed regions, or are deeply buried in the protein are expected to incur the greatest fitness costs. Disorder scores and solvent accessibility have been compared with cross-sectional diversity by Li et al. (2015). We correlated these *in* *silico* derived scores with intra-patient diversity, finding rank correlation coefficients of about 0.2–0.4 for disorder scores and solvent accessibility. While highly statistically significant, the fraction of variation in diversity explained by these scores is low, which is consistent with previous observations by Meyer and Wilke (2015). By far the best correlate of fitness cost is a cross-sectional conservation, see Table 1.

**Table 1.** Correlates of fitness cost.

gene	group M	subtype B	disorder	accessibility	RNA
*gag*	−0.51	−0.59	−0.23	−0.26	0.13
*pol*	−0.56	−0.59	−0.13	−0.31	0.09
*nef*	−0.54	−0.59	−0.30	−0.19	0.11
*env*	−0.47	−0.46	0.00	0.07	0.09
*vif*	−0.57	−0.69	−0.08	−0.16	0.06

Spearman’s rank correlation coefficients of fitness cost estimates with cross-sectional diversity (measured as entropy in group M and subtype B alignments), disorder scores, and solvent accessibility values obtained from Li et al. (2015). The column “RNA” contains rank correlation coefficients of fitness at synonymous mutations with the pairing probability predicted by Siegfried et al. (2014). Supplementary Fig. S4 shows how intra-patient/global diversity correlations improve when basing intra-patient estimates on larger numbers of patients

The distribution of fitness costs depends strongly on the consensus amino acid. Mutations of cysteins (C), histidines (H), prolines (P), tryptophans (W), and tyrosines (Y) tend to be very costly, while mutations of glutamic acid (E), lysine (K), aspartic acid (D) and arginine (R) are in average less deleterious. These patterns are consistent in *gag*, *pol*, and *env*, see Supplementary Fig. S7.

### 3.9 Most drug resistance mutations have a large fitness cost

Of particular interest are the fitness costs of mutations that confer resistance against antiretroviral drugs. The most commonly administered drugs are nucleoside analog reverse transcriptase inhibitors (NRTIs), non-nucleoside analog reverse transcriptase inhibitors (NNRTIs), protease inhibitors (PIs), and integrase inhibitors (INIs). Resistance mutations against these drugs are well known (Johnson et al. 2011).

**Figure 6.**
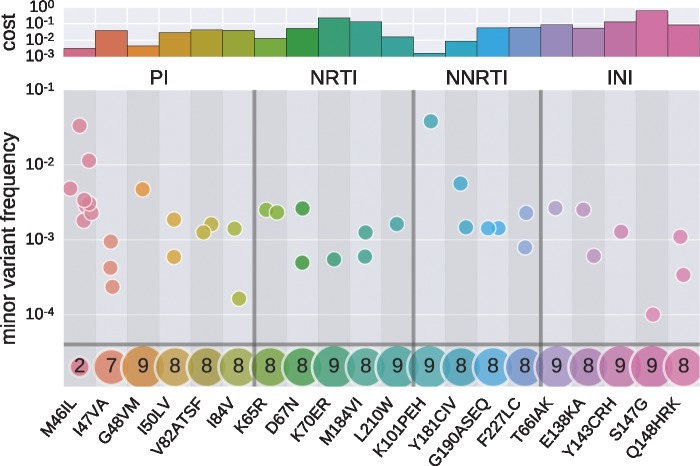
Pre-existing drug resistance mutations carry a high cost. Each point shows the average frequency of minor amino acids in individual patients. The bottom row indicates in how many out of ten patients each mutation is not observed, the top panel shows the estimated fitness costs associated with the mutations. The following mutations were never found at frequencies above 0.1 percent in any patient, indicating a large fitness cost: PI: L24I, V32I, I154VTAM, L76V, N88S, L90M; NRTI: M41L, K70ER, L74VI, Y115F, T215YF, K219QE; NNRTI: L100I, K103N, V106AM, E138K, V179DEF, Y188LCH, M230L; INI: E92Q, N155H. Most mutations are observed in no patient or only in a few patients, indicating high fitness costs.

Pre-existing low-frequency drug-resistance mutations have been associated with failing therapy (Johnson et al. 2008; Li et al. 2011). Some deep-sequencing studies have characterized such pre-existing variation in treatment-naive patients and found that drug-resistance mutations are usually below the detection limit, suggesting relatively high fitness costs (Hedskog et al. 2010; Gianella et al. 2011; Li et al. 2011). Figure 6 shows average frequencies of several drug resistance mutations in our ten patients. The majority of mutations are not seen at all, while most of the remainder is observed in only one or two patients. Only the protease mutation M46I is observed consistently across several patients. Note that the costs of very deleterious mutations might be poorly estimated if the mutations are only observed in a small number of patients. For instance, G48VM in the protease and K101PEH in the reverse transcriptase are attributed a low cost but are only observed in one patient, so their actual cost might be larger.

## 4. Discussion

Sequence evolution of HIV-1 is determined by the rate and spectrum of mutations as well as their phenotypic effects. Many studies have focused on beneficial mutations that sweep across the intra-patient HIV-1 population usually as a result of immune selection or development of drug resistance (Asquith et al. 2006; Neher and Leitner 2010; Ganusov et al. 2011; Kessinger et al. 2013). Here, we focus on neutral and deleterious mutations (the majority of all mutations). Deleterious mutations stay at low frequencies within hosts because selection constantly prunes them from the population to maintain function. Nevertheless, deleterious mutations contribute substantially to sequence evolution due to their large number: if 5,000 sites accumulate deleterious variation at frequencies of 1 percent, the typical HIV-1 genome will contain fifty such mutations.

Our estimates of HIV-1 mutation rates (Fig. 1C) are consistent with the mutation rates of HIV-1 measured in cell culture using *lacZ* assays (Mansky and Temin 1995; Abram et al. 2010), see Supplementary Fig. S1. This agreement suggests that the mutation rate of HIV-1, which is the joint rate of the HIV-1 RT, mutagenesis by the innate immune system, and the human DNA-dependent RNA polymerase II, is largely independent of cell type, despite minor differences (Holtz and Mansky 2013). To obtain sufficient statistics, we had to average the mutation rate across many sites; it will be interesting to extend these methods to individual sites and study the dependence of mutation rates on the local sequence context (Abbotts et al. 1993; Lewis et al. 1999).

While consistent with cell culture estimates, the rates that we estimate are incompatible with those reported by Cuevas et al. (2015). Whereas we measure mutations in the population of RNA virions, Cuevas et al. (2015) counted nonsense mutations in proviral DNA integrated into host cell genomes and estimated a rate of 4 × 10^−3^ per site and replication—more than 100 times higher than our estimate. Unlike in circulating viral RNA, a large fraction of proviral HIV DNA is hypermutated by enzymes of the APOBEC family (Malim 2009). Although APOBEC might partially explain the high G→A rate we found, hypermutation is approximately an all-or-nothing phenomenon in which either a sequence contains dozens of stop codons or none (Armitage et al. 2012; Cuevas et al. 2015; Delviks-Frankenberry et al. 2016). Because of this bimodal nature, hypermutation and reverse transcriptase mutation cannot be meaningfully described by a single mutation rate matrix. In the former case, a sequence with dozens of stops integrates into the host genome as an intert defective provirus, in the latter case rare independent mutations (about 0.2 per genome) can lead to gradual evolution and adaptation. Sporadic deamination by APOBEG enzymes might still contribute to the G→A mutation rate and is included in our estimate, but most hypermutated sequences are likely defective and make a minor contribution to genetic diversity, as also argued by others (Armitage et al. 2012; Delviks-Frankenberry et al. 2016).

Furthermore, proviral HIV DNA is enriched for hypermutated sequences. While productive infection rapidly leads to death of the infected cell, hypermutated proviruses tend to accumulate in HIV-1 target cells and are only removed as a result of normal cell turnover. This accumulation likely results in a multi-fold overrepresentation of hypermutated sequences compared with the probability at which hypermutation happens in a single replication cycle. Our estimates based on plasma HIV-1 RNA sequences are not affected by the accumulation of hypermutated sequences. Similarly, latently integrated sequences are unlikely to make a substantial contribution to the mutation rate estimate, since the fraction of virus that derives from the latent reservoir is small during untreated infection and these viruses tend to be similar to the replicating virus population (Wei et al. 1995; Brodin et al. 2016).

Using our time-calibrated mutation rate estimates, we then estimated absolute fitness costs from mutation selection balance. The distribution of fitness costs is consistent with those found in other viruses, where typically about 20–40 percent of mutations are lethal and another approximately 30 percent are strongly deleterious (Sanjuán 2010). We also quantified the relationship between global group M diversity (measured as entropy) and logarithmic fitness cost and found it to be approximately linear. Overall, fitness costs explain about half of the diversity in global alignments of HIV-1 sequences, while a fraction of the remainder might be linked to patient-specific processes such as immune escape or difference between viral fitness within and between patients. In addition, variation of the mutation rate along the genome and noise in our estimates surely contribute to the unexplained variation.

Several features of the HIV-1 genome, including regulatory elements at the RNA level, leave clear signatures in the fitness landscape. Constraints on synonymous mutations appear to be stronger and more prevalent in *env* than in *gag* or *pol*, consistent with earlier results that many synonymous mutations in gp120 tend to be weakly deleterious (Zanini and Neher 2013) and that *env* recoding results in non-infectious virus (Vabret et al. 2014). However, comparison of our fitness cost estimates with genome wide RNA structure predictions by Siegfried et al. (2014) and Sükösd et al. (2015) show little correlation outside of known conserved structures (see Supplementary Fig. S9 and Table 1). This absence of correlation with RNA structure is consistent with the observation that (predicted) pairing patterns evolve rapidly in most of the genome (Pollom et al. 2013) or might reflect inaccuracies in RNA structure prediction: only a minority of pairings agree between the predictions by Siegfried et al. (2014) and Sükösd et al. (2015).

Several groups have estimated fitness costs within HIV-1 proteins using experimental approaches (Martinez-Picado and Martinez 2008; Thyagarajan and Bloom 2014; Rihn et al. 2015). Our estimates presented here are complementary to those studies in two ways (see Supplementary Fig. S8). First, because of the short but dense temporal sampling, cell culture experiments are sensitive to large fitness costs, typically above 5 percent, while estimates from natural variation are most accurate for effects below a few percent. Second, *in vivo* estimates are not affected by the specific conditions of cell culture systems. Deep mutational scanning of HIV-1 proteins might overcome many of the limitations of the current experimental approaches (Haddox et al. 2016).

Computational methods to estimate fitness landscapes from cross-sectional data have also been proposed (Dahirel et al. 2011; Ferguson et al. 2013), including a recent effort to include intra-patient diversity via shallow sequencing (Hartl et al. 2016). The relationship between fitness cost and diversity, however, might be blurred since a site that is costly to mutate might still be globally diverse due to escape from CTL pressure exerted by a high-prevalence HLA allele. Indeed, we have shown in Fig. 5 that globally polymorphic sites that we estimate to have high fitness costs are overrepresented among sites known to be HLA-associated (Carlson et al. 2012). Barton et al. (2016) have shown that the rate of CTL escape depends on fitness costs. More generally, the cross-sectional inferences and our intra-patient inferences reinforce the notion that HIV-1 evolution is governed by a fitness landscape that consists of a universal component determining the replicative capacity of the virus plus a host-specific component responsible for escape mutations (Shekhar et al. 2013). Our approach based on longitudinal deep intra-patient data allows to explicitly disentangle these two contributions, since we can condition on the founder sequence and the absence of host-specific selective sweeps. Purely cross-sectional inferences of the fitness landscape likely underestimate the fitness cost of mutations at HLA-associated positions.

The frequency of drug resistance mutations is expected to be inversely proportional to their fitness cost in absence of treatment; some of these costs have been measured in cell cultures (see e.g. Chow et al. 1993; Cong et al. 2007; Martinez-Picado and Martinez 2008). Many resistance mutations quickly revert upon treatment interruption suggesting high fitness costs (Deeks 2003; Joos et al. 2008; Hedskog et al. 2010). Indeed, for most drug-resistance mutations, we estimate fitness costs in excess of 5 percent (sites where minor variation is not or only sporadically observed), see top panel in Fig. 6.

In the future, as whole genome deep sequencing becomes more common, estimates of mutation rates and the fitness landscape could be extended to a higher number of samples and other viruses. In particular, because the dataset used in this article is mostly from subtype B, deeper sampling of other HIV clades could help define the degree of universality of the HIV-1 fitness landscape. A much larger sample pool might allow site-specific inference of the mutation rates. Furthermore, by providing more accurate minor SNV frequencies, estimates of their associated fitness costs will improve, leading to a deeper understanding of the selective forces that shape viral evolution.

## Supplementary data

Supplementary data are available at *Virus Evolution* online.

## Supplementary Material

Supplementary Data
