# Supplementary material for: In vivo mutation rates and the landscape of fitness costs of HIV-1
Source: Virus Evol. 2017 Mar 2;3(1):vex003. doi: 10.1093/ve/vex003 (PMC5399928; doi:10.1093/ve/vex003)
Supplement: Supplementary Data [file vex003_Supp.pdf]

SUPPLEMENTARY MATERIAL

Patient	Gender	Transmission route	Subtype	Age* [years]	Fiebig stage*	BED* [ODn]	No. of samples	First sample [days]	Last sample [years]	HLA type A	HLA type B	HLA type C
p1	F	HET	01_AE	37	IV	0.41	12	49	8.0	02/02	08/15	03/06
p2	M	MSM	B	32	V	0.17	6	74	5.5	01/24	08/39	07/12
p3	M	MSM	B	52	VI	0.89	10	104	8.3	02/11	15/44	03/16
p5	M	MSM	B	38	III-IV	n.a.	7	132	5.9	03/33	14/58	03/08
p6	M	HET	C	31	IV	0.29	7	46	7.0	02/02	44/51	05/16
p7	M	MSM	B	31	VI	0.95	11	1905	16	02/02	15/27	01/03
p8	M	MSM	B	35	V	0.15	7	64	6.0	03/32	07/40	02/07
p9	M	MSM	B	32	VI	0.27	8	106	8.1	25/32	07/44	04/07
p10	M	MSM	B	34	II	0.10	9	18	6.1	32/32	44/50	06/16
p11	M	MSM	B	53	VI	1.22	7	167	5.5	02/32	39/44	05/12

TABLE S1 Summary of patient characteristics (adapted from Zanini *et al.* (2015)). Sample times from estimated date of infection. *, at base line; MSM, men who have sex with men; HET, heterosexual.

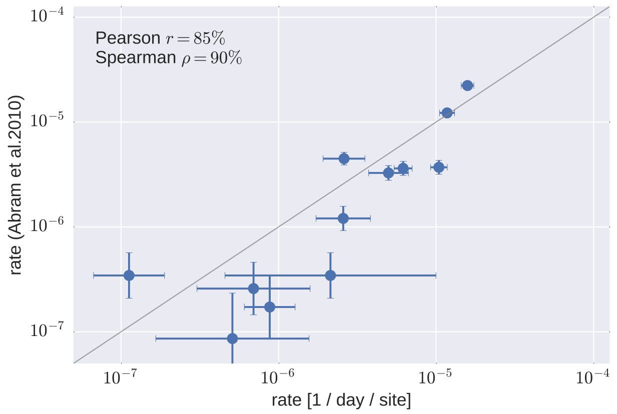

FIG. S1 Comparison of our estimates for the neutral mutation rates to *in vitro* estimates by Abram *et al.* (2010). Error bars for the estimates are standard deviations over 100 patient bootstraps. Error bars for the values from Abram et al. (2010) are standard deviations of binomial sampling noise (low-frequency mutations were observed only 1-2 times in that study).

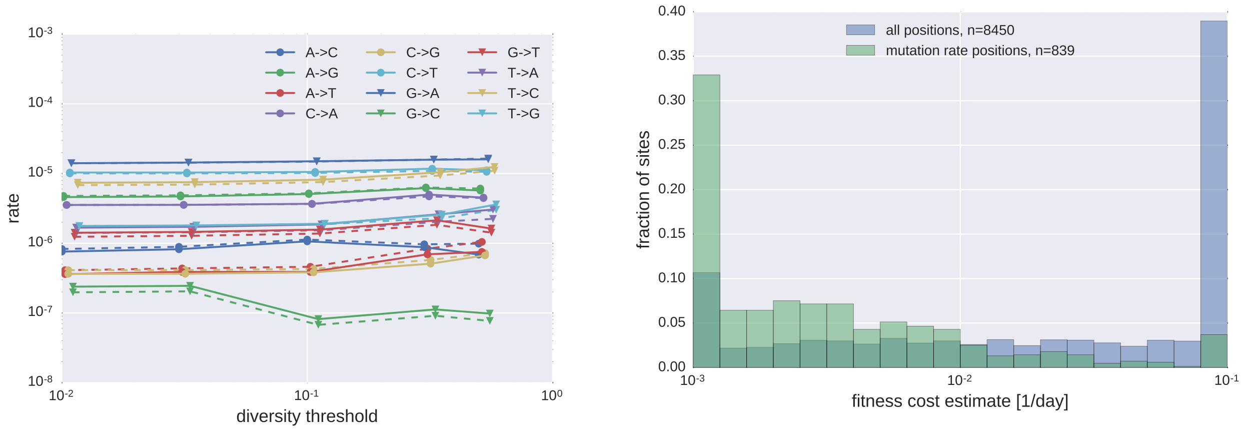

FIG. S2 Sensitivity of mutation rate estimates to the criteria used to define the set of approximately neutral positions. (A) Mutation rate estimates depend only weakly on the threshold used to define the approximately neutral set of positions or whether *gp120* is included (solid lines) or not (dashed lines). (B) The positions chosen to estimate the neutral mutation rate are among the most neutral positions as estimated by the intra-patient saturation frequencies. Note that frequencies of neutral mutations don't saturate and can be less diverse than expected due to linked selection and drift; this is not a problem for our estimates as we do not infer site-specific mutation rates.

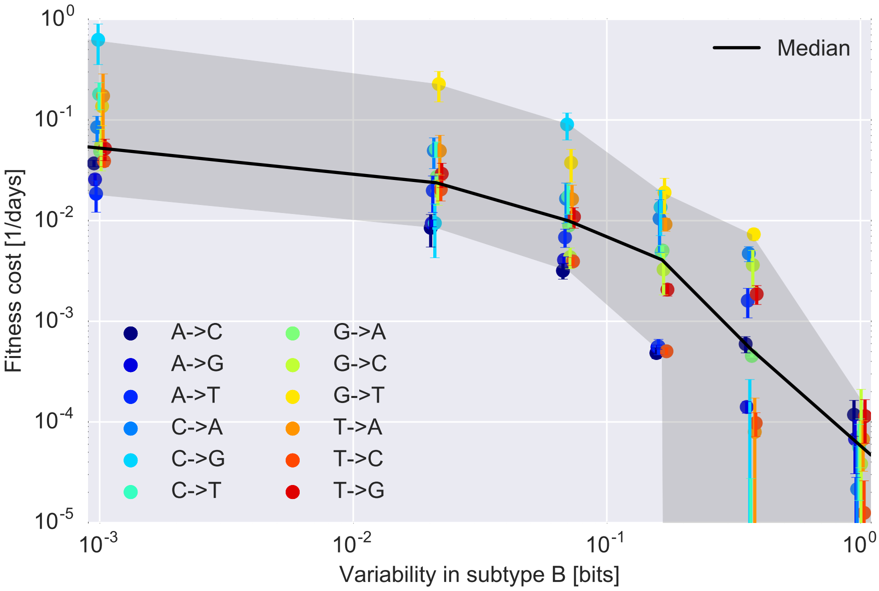

FIG. S3 Fitness cost estimates as a function of conservation in subtype B. This analysis in analogous to Fig. 2 "Sat" but we estimated separated for each of the 12 mutations. The general picture is the same as in Fig. 2, but some mutations appear to be slightly more or less suppressed than the average. The most conserved bin of genomic sites is not expected to be accurate using the "Sat" method because saturation happens too fast (see dashed dark blue line of Fig. 2A).

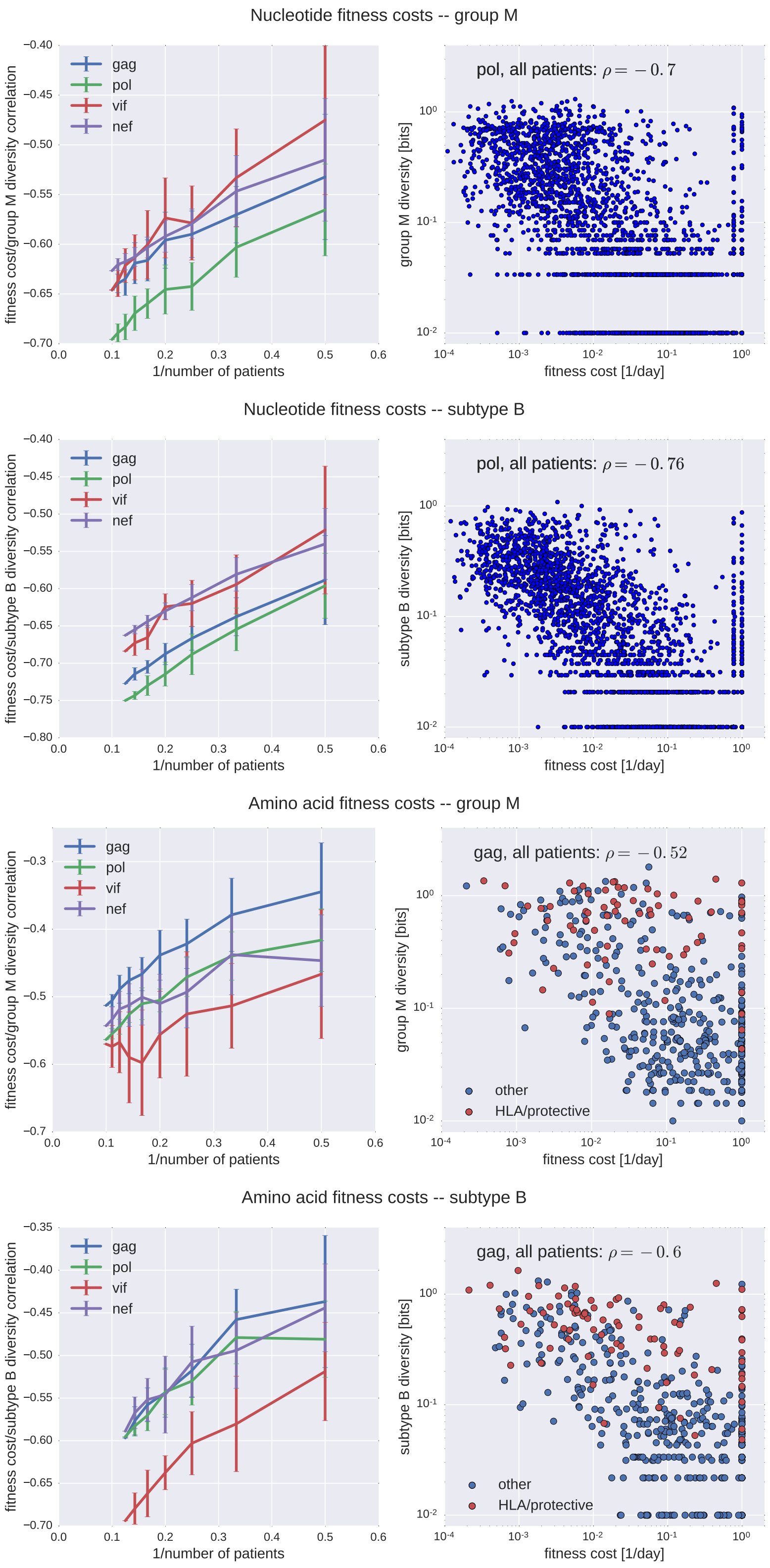

FIG. S4 **Correlation of fitness cost with global cross-sectional diversity.** The left panels show how correlation improves as fitness costs are estimates using data from more and more patients. The right panels show a scatter plot of fitness cost vs cross-sectional diversity using data from all patients for one of the proteins. The top panels show costs for nucleotide mutations, the bottom panels for amino acid mutations (and highlight HLA associated or protective sites, (Bartha *et al.*, 2013; Carlson *et al.*, 2012)).

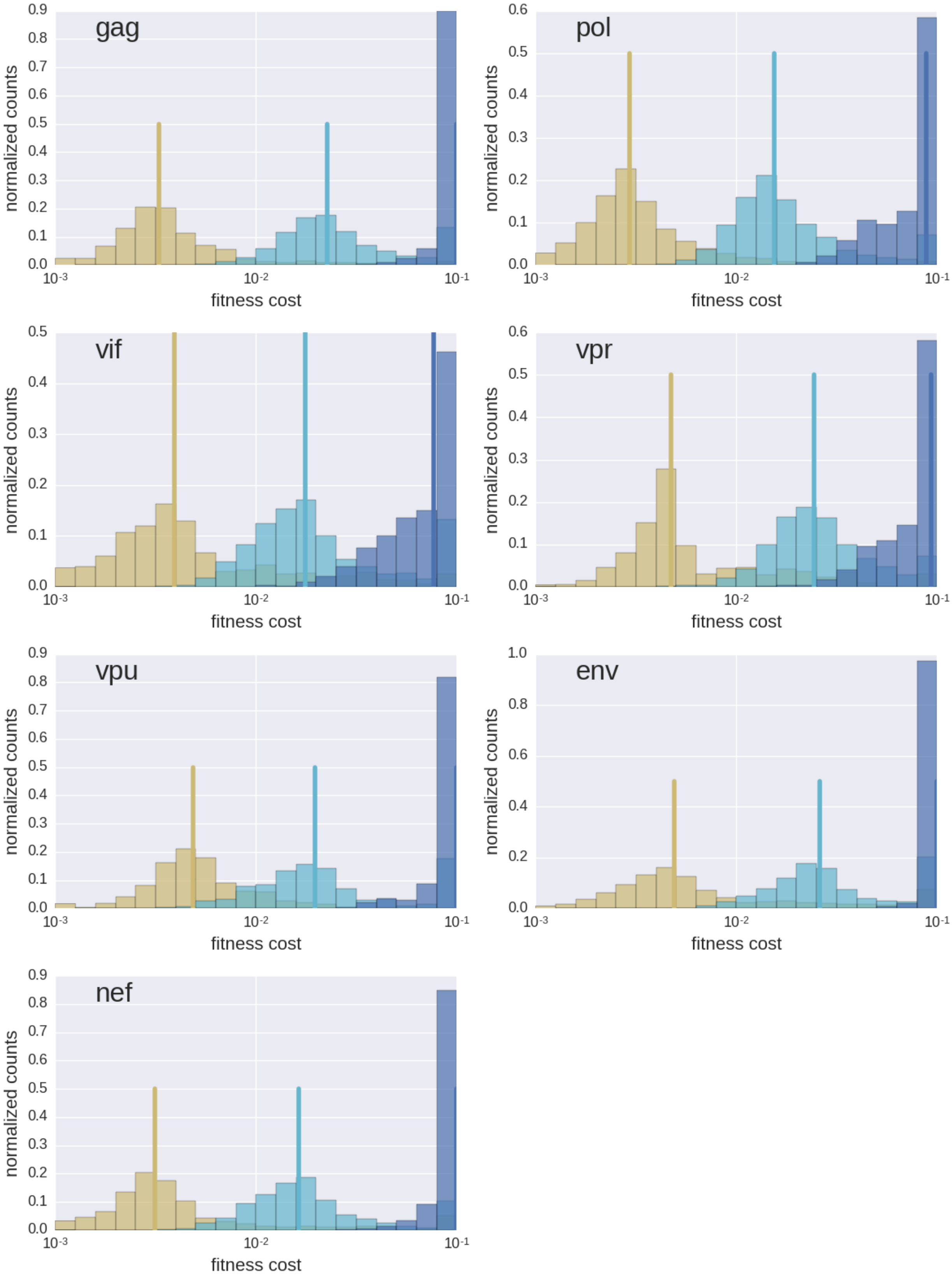

FIG. S5 **Uncertainty of the fitness costs estimates** for various regions of the genome, one region per panel. To estimate the uncertainty of our fitness cost estimates, we selected sites with fitness estimates in narrow windows indicated by the vertical lines. We then reestimated the fitness costs of these sites in 100 bootstrap resamplings of the 10 patients. The resulting distributions of fitness cost estimates approximate the uncertainty of the original estimates and are shown as histograms in the same color as the vertical lines. In most cases, the bootstrap distributions are contained within two-fold of the original estimate.

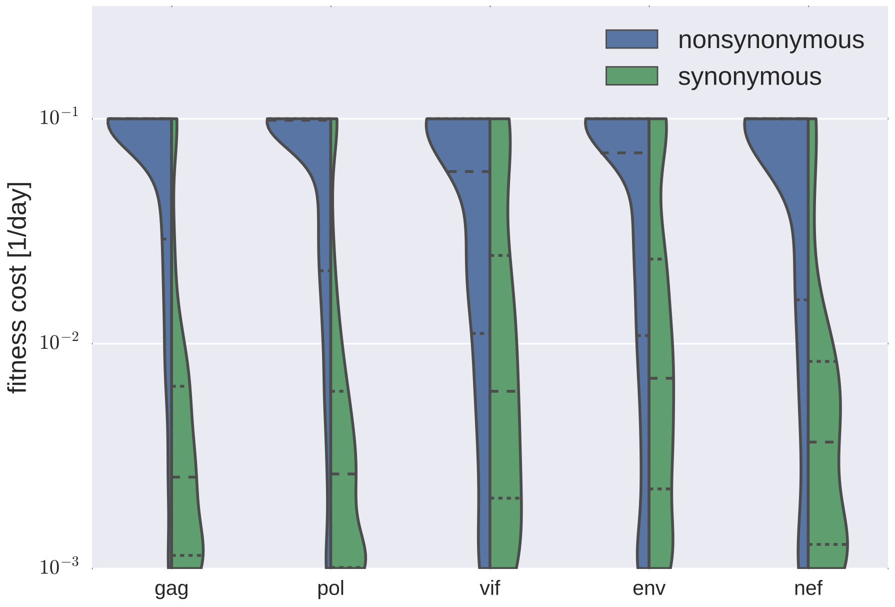

FIG. S6 **Fitness costs in different genes.** Distribution of fitness costs of synonymous and non-synonymous mutations in different genes. Note that estimates in *gp120* are expected to be less accurate due to consistent difficulties amplifying this part of the genome.

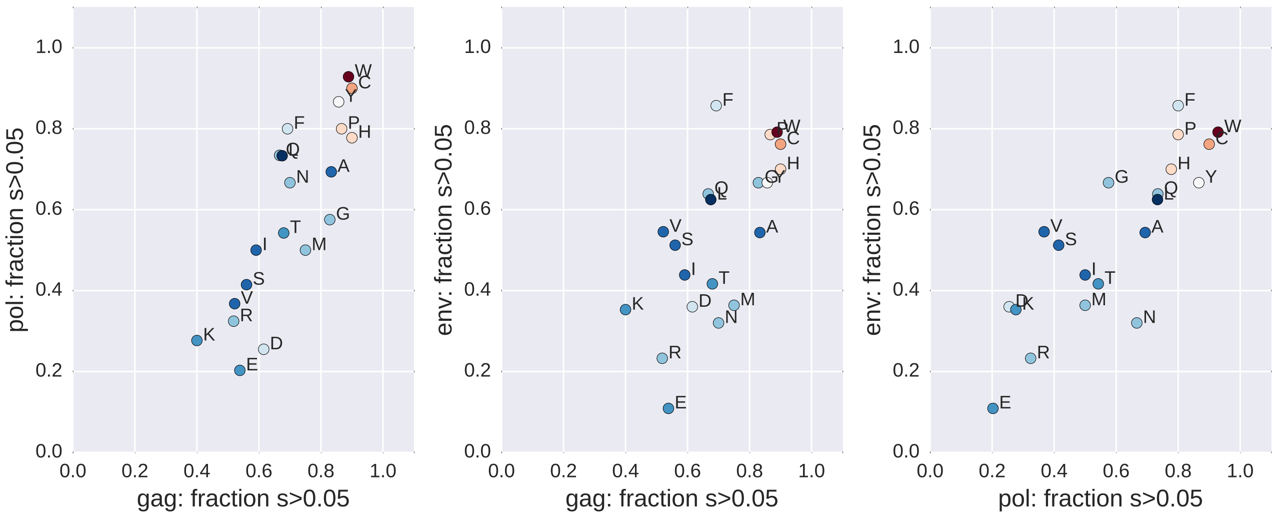

FIG. S7 **Fitness costs and consensus amino acids.** The fraction of sites with fitness costs > 0.05 per day depends consistently on the consensus amino acid. Mutations of cysteins (C), histidines (H), prolines (P), tryptophans (W), and tyrosines (Y) tend to be most costly. Points are colored according to the diagonal of the BLOSSUM80 matrix, from blue to white to red, indicating a fair degree of agreement, especially for the most costly residues.

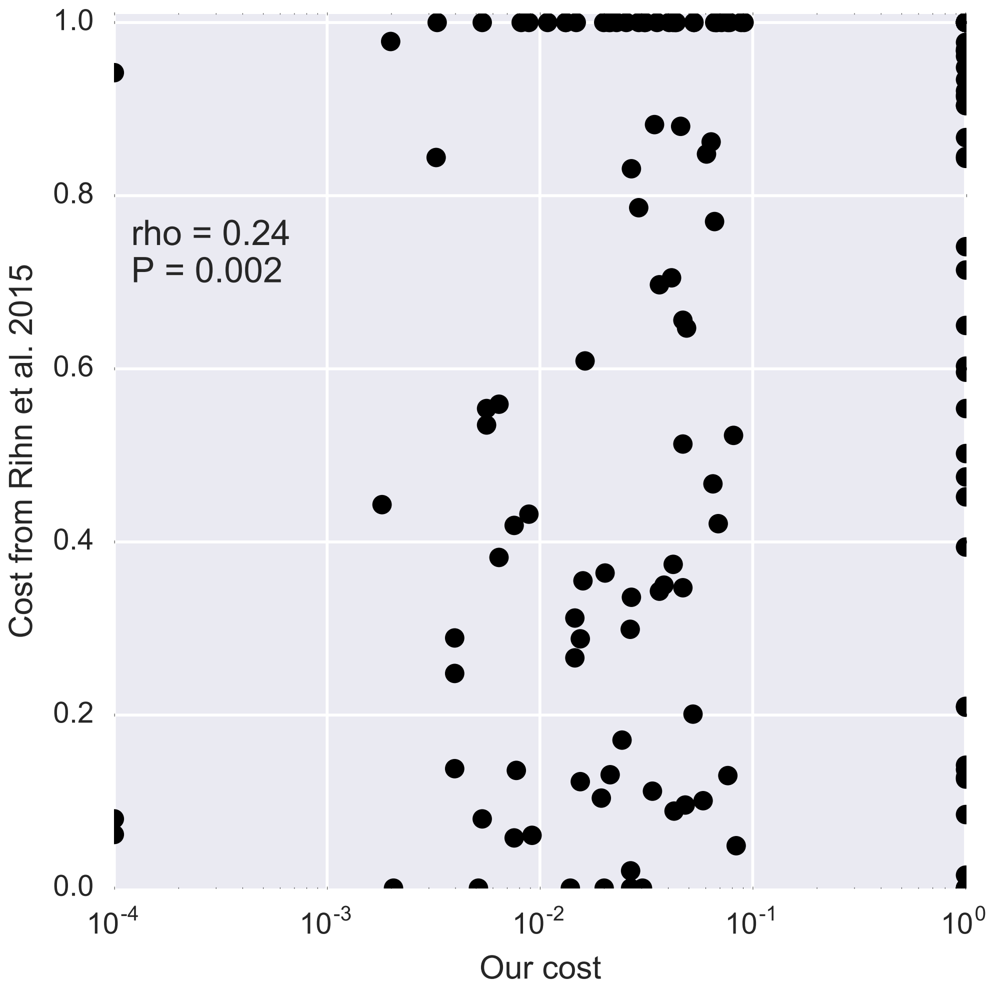

FIG. S8 Our fitness costs estimates in integrase are correlated with results from published *in vitro* experiments (Rihn *et al.*, 2015). The rank correlation coefficient is 0.24 (P-value = 0.002), which indicates a significant but low agreement between our results and Rihn et al. (2015). There are three reasons why no perfect correlation is expected. First, cell culture fitness determinations are sensitive to costs above 3-5% whereas our *in vivo* method is accurate between 0.1% and 10% approximately. This makes the two approaches nicely complementary in scope. Second, cell cultures are not perfect models of the viral dynamics in a patient, hence some selective pressures might differ. Third, one limitation of our study is that for each site we do not test for a specific mutation, so a few discrepancies might be due to this methodological difference. In cases when Rihn *et al.* (2015) tested more than one mutation at a site, the same cost from our table was reused. To further test the significance of the correlation, we repeated the correlation analysis several times after reshuffling sites and costs and found no significant correlation in those cases.

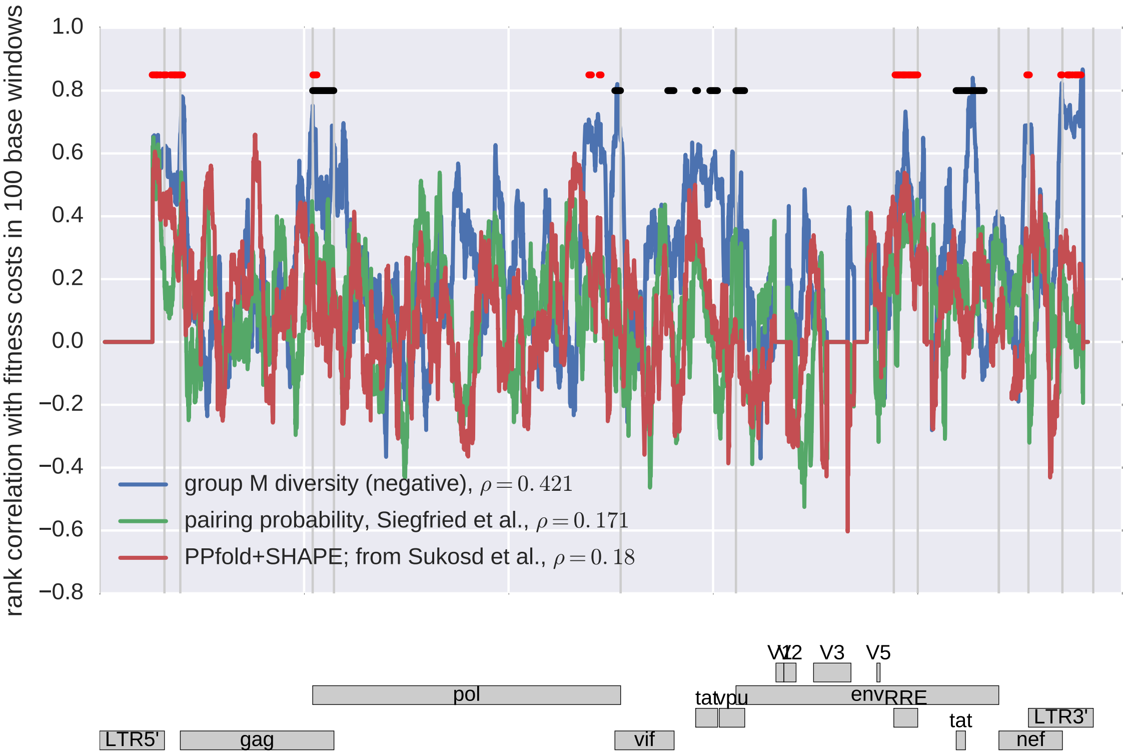

FIG. S9 Fitness estimates at synonymous* sites are well correlated (in sliding 100 bp windows) with group M diversity**, but correlation with RNA structure prediction by Siegfried *et al.* (2014) and Sükösd *et al.* (2015) is weaker and limited to a few regions. Pronounced peaks of the correlation between diversity and fitness costs at synonymous positions coincide with overlapping reading frames (marked in black in the top part of the figure) and known regulatory elements (marked in red). The strongest correlation is observed in the central and 3' poly purine tracts, around the overlap of *gag* and *pol*, and in the 3' LTR. The genome wide correlation (given in the legend) is highly significant in all cases but low for RNA structure predictions. *Synonymous sites are defined here as those at which the transition does not result in in an amino acid change in *gag*, *pol*, *vif*, *vpu*, *env*, and *nef*. **The graph reports the negative correlation with group M diversity such that the null expetation is positive correlation in all cases.

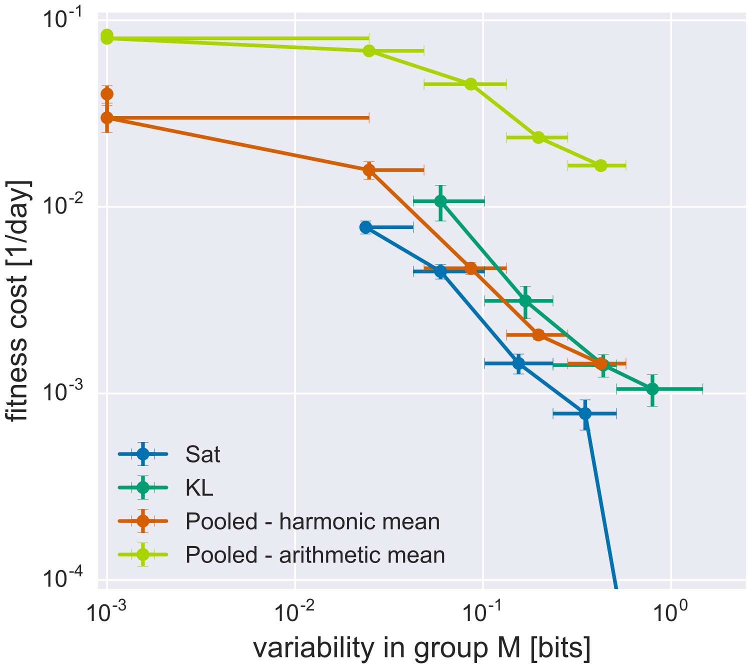

FIG. S10 Fitness cost estimates using a probabilistic model of mutation trajectories ("KL", see below) are consistent with the estimates from the saturation behavior of average trajectories ("Sat") and single site estimates ("Pooled harmonic mean"). The "Sat" and "Pooled harmonic mean" curves are the same as in Fig. 2, the "KL" curve uses the estimate method based on minimization of Kullback-Leibler divergence (see below). The "Sat" estimate averages mutation frequencies, which saturate at levels inversely proportional to the fitness costs. Hence this method essentially estimates a harmonic mean of selection coefficients and agrees well with the harmonic mean of site specific estimates ("Pooled"). The harmonic mean is dominated by the smallest selection coefficients in group. In contrast the arithmetic mean of fitness costs is dominated by the largest values and is much larger than the harmonic mean. This spread reflects the diversity of fitness costs among the sites used for averaging within each conservation group.

Estimation of selection coefficients by Kullback-Leibler divergence minimization

In addition to the two modelling methods presented in Fig. 2, "Sat" and "Pooled", we tested a third approach that exploits the time information of samples (like the "Sat" method) but also models the temporal correlations of SNV frequencies (see Fig. S10). These correlations are not accounted for in the "Sat" fitting procedure which simply fits average values for each bin.

We capture the correlation structure of the SNV frequency trajectories by modelling the full probability distribution $P(\mathbf{x})$ of observing all SNVs from all times at a certain combination of frequencies:

$$\mathbf{x} = (x_{t,i} \dots),$$

where t indicates each time point and i each conservation group. We combine all SNP trajectories (summed minor derived states) of all sites within one conservation group into $\mathbf{x}$, separately for each patient. We approximate the joint probability distribution $P(\mathbf{x})$ by a theoretical distribution $W(\mathbf{x})$ that is the solution of the stochastic equation (1) with a constant diffusive noise term $\eta(t)$ to make it mathematically tractable

$$\left\langle \eta^2(t) \right\rangle \propto Dt.$$

where D defines the noise intensity. The solution of eq. (1) under these simplifying assumptions is a multivariate Gaussian distribution:

$$W(\mathbf{x}) = \frac{\exp\left[-\frac{1}{2}(\mathbf{x} - \langle \mathbf{x} \rangle)^T K^{-1} (\mathbf{x} - \langle \mathbf{x} \rangle)\right]}{\sqrt{(2\pi)^N \det K}}, \tag{6}$$

where K is the covariance matrix of SNP frequencies. Mean and covariance of $W(\mathbf{x})$ are given respectively by

$$\begin{aligned} \langle x(t) \rangle &= \frac{\mu}{s}\left(1 - e^{-st}\right), \\ K(t, t') &= \frac{D}{s}\left[e^{-s|t-t'|} - e^{-s(t+t')}\right], \end{aligned} \tag{7}$$

We now want to estimate the parameters s and D from the data while keeping μ, the mutation rate, fixed at the measured value $1.2 \cdot 10^{-5}$ per day per site. To this end, we construct an empirical distribution of SNP frequency trajectories as a multivariate Gaussian with mean and covariances obtained by averaging the data across sites:

$$\begin{aligned} \hat{x}(t) &= \frac{1}{L}\sum_k x_k(t), \\ \kappa(t_i, t_j) &= \frac{1}{L-1}\sum_k \left[x_k(t_i) - \hat{x}(t_i)\right]\left[x_k(t_j) - \hat{x}(t_j)\right]. \end{aligned} \tag{8}$$

Here k is the site/position index, the $\hat{x}$ designates average minor SNV frequency in the conservation group analysed, t_i and t_j are time points along the trajectory, and L is the number of sites used in the average.

Mean and covariance fully determine the empirical Gaussian distribution, so we can extract the best model parameters by minimizing the distance of this distribution and the theoretical one. A convenient measure of the divergence between the two distributions is so-called Kullback-Leibler divergence, defined as

$$KL = \int P(\mathbf{x}) \log\left[\frac{P(\mathbf{x})}{W(\mathbf{x})}\right] d\mathbf{x}. \tag{9}$$

Averaging over the empirical distribution $P(\mathbf{x})$ is now equivalent to averaging over sites, which allows us to write the Kullback-Leibler divergence (KL) as

$$\begin{aligned} KL = C - \frac{1}{L}\log W(\mathbf{x}) &= C + \log\sqrt{(2\pi)^N \det K} \\ &+ \frac{1}{2}\sum_{i,j}\left\{\left[\hat{x}(t_i) - \langle x(t_i)\rangle\right](K^{-1})_{ij}\left[\hat{x}(t_j) - \langle x(t_j)\rangle\right] + (K^{-1})_{ij}\kappa_{ji}\right\}. \end{aligned} \tag{10}$$

Finally, we notice that for different conservation groups, the KL is additive. We can thus sum over all conservation groups to estimate all s and D parameters simultaneously (one s and one D per group). The resulting values for s are shown in Fig. S10 as the "KL" curve and is in good agreement with the two previous methods used to estimate average fitness costs.
